# Gene silencing of *Nox4* by CpG island methylation during hepatocarcinogenesis in rats

**DOI:** 10.1242/bio.020370

**Published:** 2016-11-28

**Authors:** Guadalupe S. López-Álvarez, Tomasz K. Wojdacz, Claudia M. García-Cuellar, Hugo C. Monroy-Ramírez, Miguel A. Rodríguez-Segura, Ruth A. Pacheco-Rivera, Carlos A. Valencia-Antúnez, Nancy Cervantes-Anaya, Ernesto Soto-Reyes, Verónica R. Vásquez-Garzón, Yesennia Sánchez-Pérez, Saúl Villa-Treviño

**Affiliations:** 1 Departamento de Biología Celular, Centro de Investigación y de Estudios Avanzados del IPN (CINVESTAV), Av. IPN No. 2508 Col. San Pedro Zacatenco, CDMX CP 07360, México; 2 Aarhus Institute of Advanced Studies andDepartment of Biomedicine, Bartholins Allé 6 Building, 1242, 8000 Aarhus C, Denmark; 3 Instituto Nacional de Cancerología (INCan), Subdirección de Investigación Básica, San Fernando No. 22, Tlalpan, CDMX CP 14080, México; 4 Departamento de Bioquímica, Escuela Nacional de Ciencias Biológicas-IPN, Carpio y Plan de Ayala S/N, Col. Casco de Santo Tomas, CDMX CP 11340, México; 5 CONACYT, Facultad de Medicina y Cirugía, Universidad Autónoma “Benito Juárez” de Oaxaca, Ex-Hacienda de Aguilera S/N Carretera a San Felipe del Agua, Oaxaca, Oax., CP 68020, México

**Keywords:** Chemical hepatocarcinogenesis, *Casp3*, *Cldn1*, *Pex11a*, *Nox4*, DNA methylation, Downregulation

## Abstract

The association between the downregulation of genes and DNA methylation in their CpG islands has been extensively studied as a mechanism that favors carcinogenesis. The objective of this study was to analyze the methylation of a set of genes selected based on their microarray expression profiles during the process of hepatocarcinogenesis. Rats were euthanized at: 24 h, 7, 11, 16 and 30 days and 5, 9, 12 and 18 months post-treatment. We evaluated the methylation status in the CpG islands of four deregulated genes (*Casp3*, *Cldn1*, *Pex11a* and *Nox4*) using methylation-sensitive high-resolution melting technology for the samples obtained from different stages of hepatocarcinogenesis. We did not observe methylation in *Casp3*, *Cldn1* or *Pex11a.* However, *Nox4* exhibited altered methylation patterns, reaching a maximum of 10%, even during the early stages of hepatocarcinogenesis. We observed downregulation of mRNA and protein of *Nox4* (97.5% and 40%, respectively) after the first carcinogenic stimulus relative to the untreated samples. Our results suggest that *Nox4* downregulation is associated with DNA methylation of the CpG island in its promoter. We propose that methylation is a mechanism that can silence the expression of *Nox4*, which could contribute to the acquisition of neoplastic characteristics during hepatocarcinogenesis in rats.

## Abstract (precis)

**Summary:** Many genes downregulated by DNA methylation are tumor suppressor genes. In the present study, DNA methylation of *Nox4* is observed, with implications for *Nox4* as a potential therapeutic target for liver or other cancers.

## INTRODUCTION

Carcinogenesis is a multifactorial event related to numerous genetic alterations and epigenetic abnormalities (Sharma et al., 2010). This process can be divided into three stages from an operational perspective: initiation, promotion and progression (Oliveira et al., 2007). To understand the mechanisms related to cancer development, rodent models have been established. Models of chemical hepatocarcinogenesis (HCG) are widely used to study the different stages of liver carcinogenesis. In HCG models, reactive oxygen species (ROS) play an important role (Sanchez-Perez et al., 2005), and the documented increase in ROS levels in cancer cells is due, in part, to increased metabolic activity (Tong et al., 2015).

Additionally, during carcinogenesis, genotoxic mechanisms can produce changes in genomic DNA that lead to mutations (Franco et al., 2008). In hepatocellular carcinoma (HCC), previous studies have reported mutations in β-catenin, overexpression of receptors (such as ErbB and MET), chromosomal gains in 1q, 6p, 8q, 17q and 20q and chromosomal losses in 1p, 4q, 8p, 13q and 17p (Farazi and DePinho, 2006; Forner et al., 2012). However, in HCG models, mutations have been found in oncogenes (such as *Nrf2* and *H-ras*), which are critical for the progression and development of liver carcinogenesis (Unterberger et al., 2014; Zavattari et al., 2015).

In contrast, non-genotoxic mechanisms do not affect DNA sequences, but they are capable of affecting gene expression via epigenetic mechanisms (Franco et al., 2008). One of the most studied epigenetic mechanisms in transcriptional regulation is the methylation of CpG dinucleotides, which are concentrated in large clusters called CpG islands. These CpG islands are mostly enriched in gene promoters from −2 kb to +1 kb relative to the transcription start site (McCabe et al., 2009).

These CpG islands are important in cancer when they display an increase or decrease in methylation, resulting in decreased or increase expression of the gene, respectively (Kulis and Esteller, 2010; McCabe et al., 2009). For example, in human liver tumors, researchers have described the deregulation of the expression of many tumor suppressor genes as a result of DNA methylation in CpG islands, including *p16^INK4a^*, *p15^INK4B^*, *GSTP1*, *RB1*, *RASSF1a*, *SOCS3*, *CDH1* and *COX-2* (Farazi and DePinho, 2006; Pogribny and Rusyn, 2014). HCG models have also revealed the methylation of genes such as *p16 ^INK4a^*, *Timp3*, *Rassf1a*, *Casp8* and *Cdh13* (Valencia Antunez et al., 2014; Yu et al., 2002).

Regarding the deregulation of genes via epigenetic mechanisms, we have previously described profound changes in gene expression during liver carcinogenesis using microarray-based gene expression profiling (GEP) (Vasquez-Garzon et al., 2015), including the significant downregulation of several genes. Because deregulation of gene expression through methylation plays an important role in the carcinogenic process, it is necessary to identify new targets that participate in liver carcinogenesis. In our GEP analysis, we found 1238 downregulated genes at different time points in an HCG model (fold-change in expression was calculated using Partek Genomic Suite software, where <−1 indicates downregulation and >1 indicates upregulation).

Based on our previous studies, we analyzed the methylation status of a set of genes – *Casp3* (caspase 3), *Cldn1* (claudin 1), *Pex11a* (peroxisomal biogenesis factor 11 alpha) and *Nox4* (NADPH oxidase 4) – during different stages of HCG. These genes are involved in the regulation of vital biological processes, including apoptosis (*Casp3*), the formation and function of tight junctions (*Cldn1*), peroxisome membrane biogenesis (*Pex11a*) and the formation of ROS (*Nox4*) (Honda et al., 2007; Schrader et al., 2012; Weyemi et al., 2013; Yakovlev et al., 2010). In the context of gene regulation by epigenetic mechanisms, *Casp3* and some claudins (CLDN3) have been reported in the maturing rat brain and in ovarian cancer cells, respectively (Honda et al., 2007; Yakovlev et al., 2010). Also, *DUOX1* and *DUOX2*, which belong to the NAPDH oxidase family of enzymes, are methylated in human lung cancer (Luxen et al., 2008).

The NADPH oxidase family has seven members: *NOX1*-*NOX5*, *DUOX1* and *DUOX2*. These enzymes are responsible for producing ROS for specific physiological functions, such as signal transduction and cell differentiation, and they are differentially expressed in tissues and display different subcellular localizations (Krause, 2004; Ushio-Fukai, 2006; Weyemi et al., 2013). *Nox4*, a gene of interest in this study, is overexpressed in a variety of human malignancies, such as pancreatic cancer, renal cell carcinoma, prostate cancer and melanoma (Kumar et al., 2008; Lee et al., 2007; Maranchie and Zhan, 2005; Yamaura et al., 2009). However, *Nox4* expression was decreased in a rat model of HCG through a mechanism that remains unknown (Liu et al., 2009). On the other hand, *Nox4* is downregulated after partial hepatectomy (PH) and in a diethylnitrosamine (DEN)-induced HCG model in mice, suggesting that *Nox4* inhibits hepatocyte proliferation in normal liver (Crosas-Molist et al., 2014).

We only observed changes in the DNA methylation pattern of the analyzed region in the CpG island of *Nox4* during HCG development. We also found an association between mRNA downregulation and the methylation status of *Nox4*. Moreover, NOX4 protein was downregulated in this model, both in preneoplastic lesions and in tumor tissue. We propose that *Nox4* downregulation by DNA methylation could favor the proliferation of cancer cells during different stages of HCG; accordingly, *Nox4* could act as a potential tumor suppressor gene in normal rat liver.

## RESULTS

### Macroscopic characterization of rat liver during HCG

**Fig. 1.**
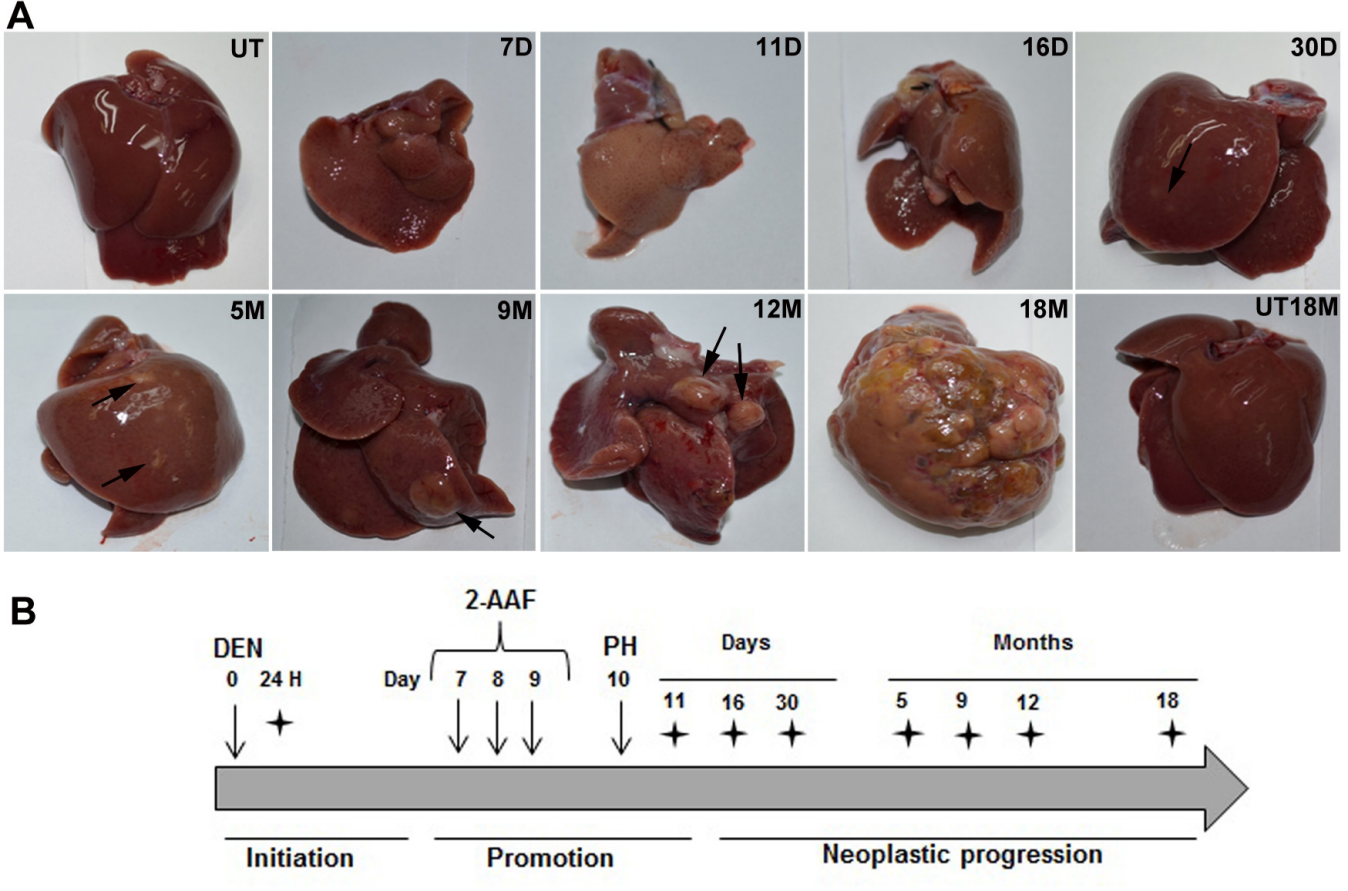
**Model of hepatocarcinogenesis in rats.** (A) Representative livers at each time point. At 7D, 11D and 16D, the livers displayed reduced size after the administration of DEN and 2-AAF and subjection to PH. Preneoplastic nodules are shown at 30D, 5M, 9M and 12M (black arrows). Tumor areas occupy most of the liver at 18M. UT livers at 0H and at 18M are shown (UT and UT 18M). (B) Fischer-344 rats were induced by an intraperitoneal injection of 200 mg/kg DEN, which was promoted by 20 mg/kg of 2-AAF after 7, 8 and 9 days. At day 10, the rats were subjected to PH. Groups of five rats were euthanized at the following time points: 24H, 7D, 11D, 16D and 30D and at 5M, 9M, 12M and 18M (black stars). The initiation, promotion and neoplastic progression stages are shown during hepatocarcinogenesis. DEN, diethylnitrosamine; 2-AAF, 2-acetylaminofluorene; PH, partial hepatectomy; UT, untreated; H, hours; D, days; M, months.

We obtained livers from untreated (UT) and treated rats at different time points after subjection to HCG. The UT livers exhibited normal features without macroscopic damage. There were five characteristic lobes in the liver of these rats (*Rattus norvegicus*), which had an average weight of 7.26 g (Fig. 1A; UT). Following the administration of DEN, 2-acetylaminofluorene (2-AAF) and PH, we observed livers of smaller sizes and weights, with an average weight of 4.2 g at 7 days and 2.74 g at 11 days (Fig. 1A; 7D, 11D) (D: days). The liver weights increased by an average of 6 g after 16 days of treatment. Finally, after 30 days of treatment, we observed livers of normal sizes and weights (average of 8 g). At 30 days post-treatment, the livers exhibited macroscopic pre-neoplastic nodules (Fig. 1A; 30D). We observed more visible preneoplastic lesions at 5, 9 and 12 months post-treatment, and we observed tumors by 18 months after treatment (Fig. 1A; 5M, 9M, 12M, 18M) (M: months). A control group of livers at 18 months without treatment exhibited normal macroscopic appearances with five lobes (Fig. 1A; UT18M).

### A set of genes are deregulated in HCG

We analyzed the deregulated genes in this model according to the relationship described between the downregulation of genes and the methylation status of CpG islands in their promoter regions. Using information obtained via microarray analyses, in accordance with the fold-change shown by the Partek Genomic Suite software for this model of HCG, we observed 1238 downregulated genes in the treated rats. Next, we screened these genes according to the following parameters:

- Genes that were downregulated greater than or equal to two fold-change after 24 h post-DEN compared with untreated rats.
- Genes with CpG islands according to the UCSC (University of California Santa Cruz) Genome Browser on Rat, accessed in March 2012.
- Canonical, biological and toxicological gene classification using Partek software.

We selected four genes that were differentially deregulated during the development of HCG: *Casp3*, *Pex11a*, *Cldn1* and *Nox4*.

**Fig. 2.**
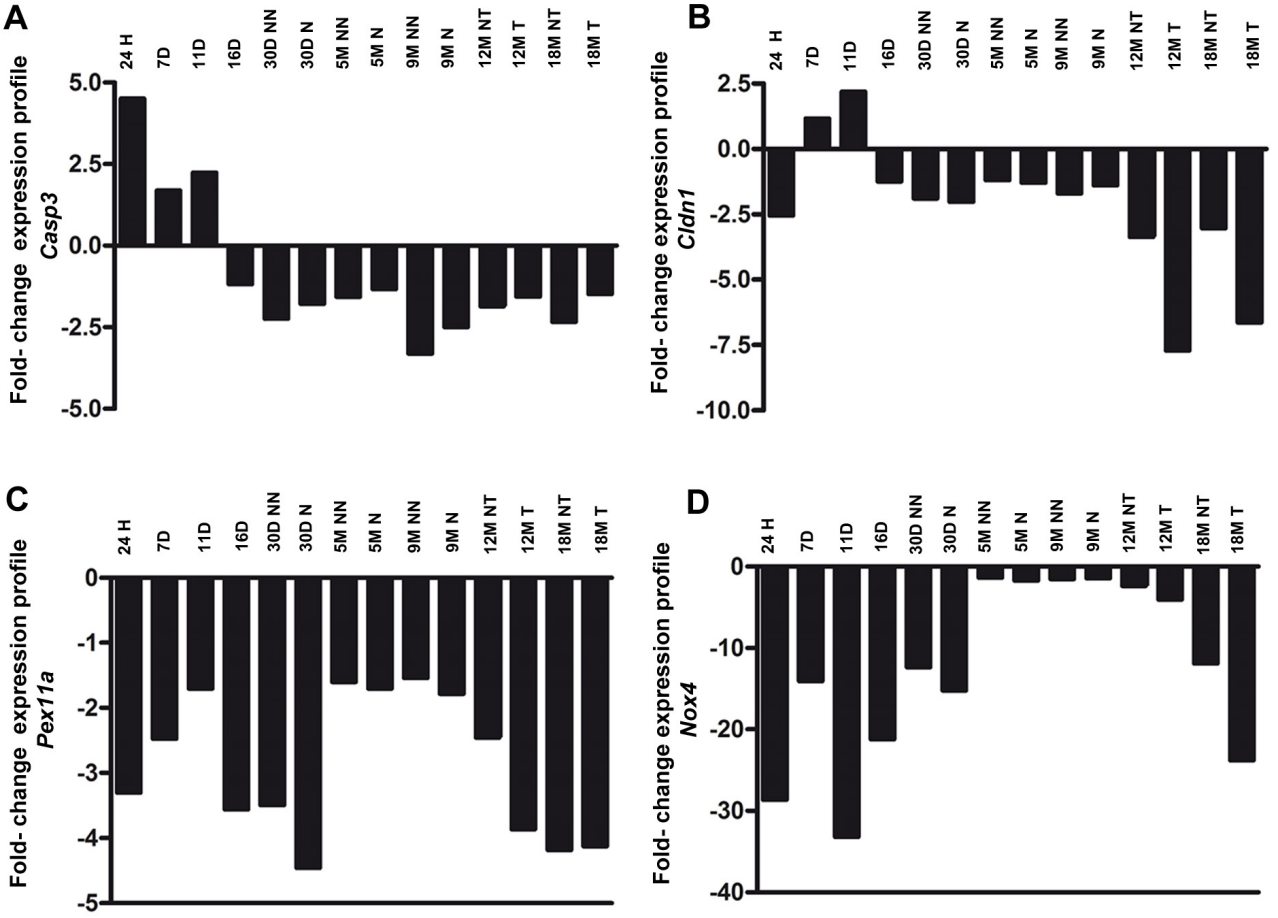
***Casp3*, *Cldn1*, *Pex11a* and *Nox4* genes are deregulated during HCG.** Fold-change in the expression levels of (A) *Casp*3, (B) *Cldn1*, (C) *Pex11a* and (D) *Nox4*, calculated using Partek Genomic Suite software. The graphs show the expression ratio between the different time points during liver carcinogenesis post-treatment (<−1 was considered downregulation and >+1 was considered upregulation). H, hours; D, days; M, months; NN, non-nodule; N, nodule; NT, non-tumor; T, tumor.

*Casp3* was upregulated at 24 h and on days 7 and 11 during the initiation and promotion stages. However, from day 16 onward, *Casp3* was downregulated during the progression stage (Fig. 2A). *Cldn1* was downregulated during the initiation stage at 24 h and was upregulated during the promotion stage, but from day 16 onward, *Cldn1* was downregulated throughout the progression stage of HCG (Fig. 2B). *Pex11a* was downregulated at every stage of HCG (Fig. 2C). The *Nox4* gene was also downregulated, displaying a 28-fold decrease during the initiation stage after the administration of DEN; this downregulation persisted during the promotion and progression stages. *Nox4* was the only gene that presented with dramatic downregulation, with minimum and maximum of 1.3-fold decrease at 5M NN (NN: non-nodule, tissue adjacent to preneoplastic lesions) and 33-fold decrease at 11D, respectively (Fig. 2D).

### The *Nox4* CpG island displays changes in DNA methylation during HCG

We found CpG islands in the promoter regions of *Casp3*, *Cldn1*, *Pex11a* and *Nox4*, which is consistent with predictions from the UCSC Genome Browser and from MethPrimer software (http://www.urogene.org/methprimer/) (Fig. S1). Therefore, we studied the methylation status in a fragment of the CpG islands in the promoters of these genes using methylation-sensitive high-resolution melting curve (MS-HRM) technology. DNA methylation was not present in the fragment of CpG islands corresponding to *Casp3*, *Cldn1* and *Pex11a* in any of the samples analyzed during the initiation, promotion and progression stages (Fig. S2). Therefore, we concluded that these genes are not likely regulated by methylation in their CpG islands during HCG.

**Fig. 3.**
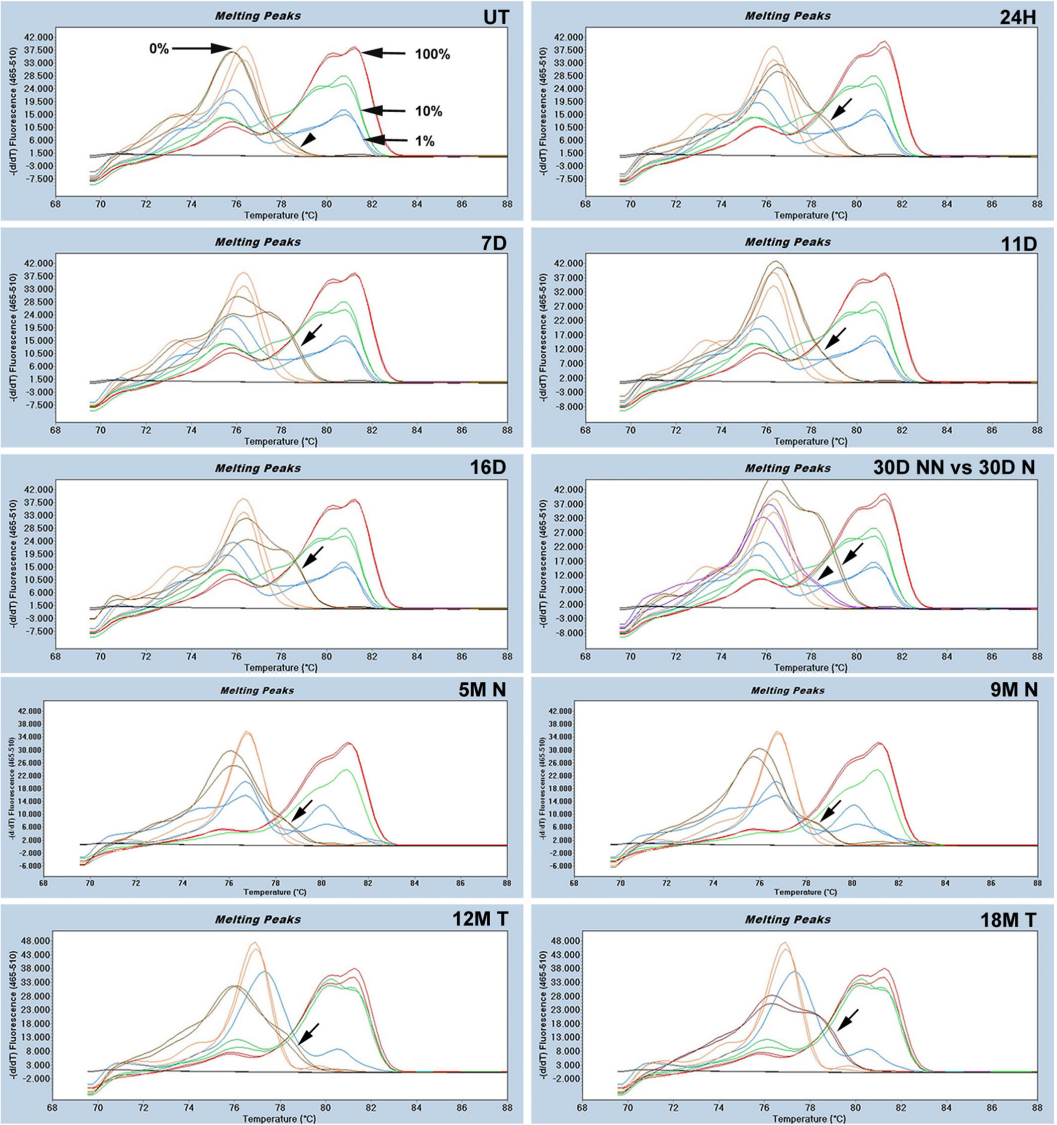
**The *Nox4* CpG island displays changes in DNA methylation patterns during HCG.** The MS-HRM profile is shown in each panel. Standard melting peaks (100% methylation, red; 10% methylation, green; 1% methylation, blue; 0% unmethylated, orange) are marked with black arrows. DNA from the UT liver shows a melting peak of 0% methylation (brown, marked with a black arrowhead). DNA peaks from 24H, 7D, 11D, 16D and 30D N samples are depicted as melting peaks between 1-10% methylation (brown, marked with black arrows). The 30D NN sample shows a melting peak corresponding to 0% methylation (purple, marked with an arrowhead). The 5M N, 9M N and 12M T display melting peaks corresponding to between 0-1% methylation (brown, marked with black arrows). The 18M T shows melting peaks corresponding to between 1-10% methylation (brown, marked with a black arrow). The melting peaks are the result of taking the negative derivative (d) of the melting curve data divided by the derivative with respect to time (dz/dT), using 480 SW 1.5 LightCycler software. H, hours; D, days; M, months; N, nodule; NN, non-nodule; T, tumor.

In contrast, the fragment of CpG island corresponding to *Nox4* showed 0% methylation in the UT livers (Fig. 3; UT) and during the initiation and promotion stages, the DNA obtained at 24H (H: hours), 7D and 11D displayed methylation levels between 1% and 10% (Fig. 3; 24H, 7D and 11D). DNA isolated at 16D and 30D N (N: nodule) (Fig. 3; 16D and 30D N) exhibited methylation levels between 1% and 10%. Interestingly, DNA from 30D NN showed 0% methylation, which suggests a difference in methylation status between the DNA from the non-nodule and the nodule area (Fig. 3; 30D NN vs 30D N). We observed a methylation level between 0-1% in the DNA samples analyzed during the progression stage for 5M N and 9M N, as well as for 12M T (T: tumor) (Fig. 3; 5M N, 9M N, 12M T), and the extent of methylation appeared to increase in the 18-month tumor samples, which exhibited methylation levels between 1 and 10% (Fig. 3; 18M T). The DNA samples obtained from the tissue adjacent to the neoplastic lesion and the tumors (non-nodules and non-tumor) at 5M, 9M, 12M and 18M showed 0% methylation (data not shown). Overall, our analyses of the MS-HRM profiles revealed low methylation in the region of the CpG Island in the *Nox4* promoter in all samples during the different stages of HCG.

**Fig. 4.**
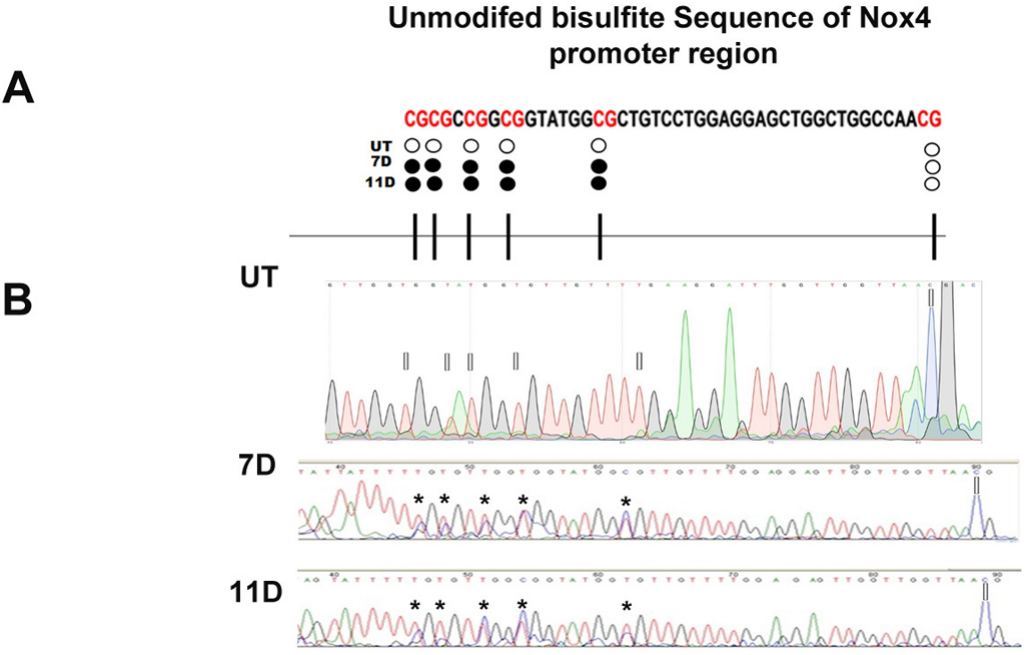
**Sequencing of DNA samples isolated during the promotion stage in HCG.** (A) Representative image of each CpG dinucleotides unmethylated (filled circles white) at UT samples and methylated dinucleotides (filled circles black) at 7 and 11 days samples in the end part of the *Nox4* island (CpG island ‘shores’). (B) Confirmation of DNA methylation as indicated by MS-HRM in 7D and 11D by sequencing during liver carcinogenesis. Unmethylated CpG sites are present as T after treatment of the DNA with sodium bisulfite in UT samples (white rectangles). Methylated CpG sites with double peaks of T (unmethylated allele) and C (methylated allele) in DNA from samples at 7D and 11D post-treatment (black asterisks). UT, untreated; D, days; C, cytosine (blue); G, guanine (black); T, thymine (red); A, adenine (green).

The MS-HRM profiles revealed the presence of methylation but do not specify the precise sites of methylation within the sequence of interest in *Nox4*; therefore, we sequenced a group of PCR products. We observed DNA methylation in the end part *Nox4* island (CpG island ‘shores’) (Fig. 4A). Samples from UT rats showed thymine peaks, indicating that the sodium bisulfite treatment changed unmethylated cytosine to thymine, confirming an unmethylated state (Fig. 4B; UT). In contrast, we observed double peaks of mixed thymine and cytosine at 7D and 11D post-treatment (Fig. 4B; 7D and 11D), confirming a state of heterogeneous methylation of samples exposed to carcinogenic treatment. Additional data in this study revealed DNA methylation levels between 10% and 50% in the human CpG Island of the *NOX4* in the HepG2 liver cancer cell line (Fig. S3).

### *Nox4* mRNA is downregulated in association with its DNA methylation status during all stages of HCG

**Fig. 5.**
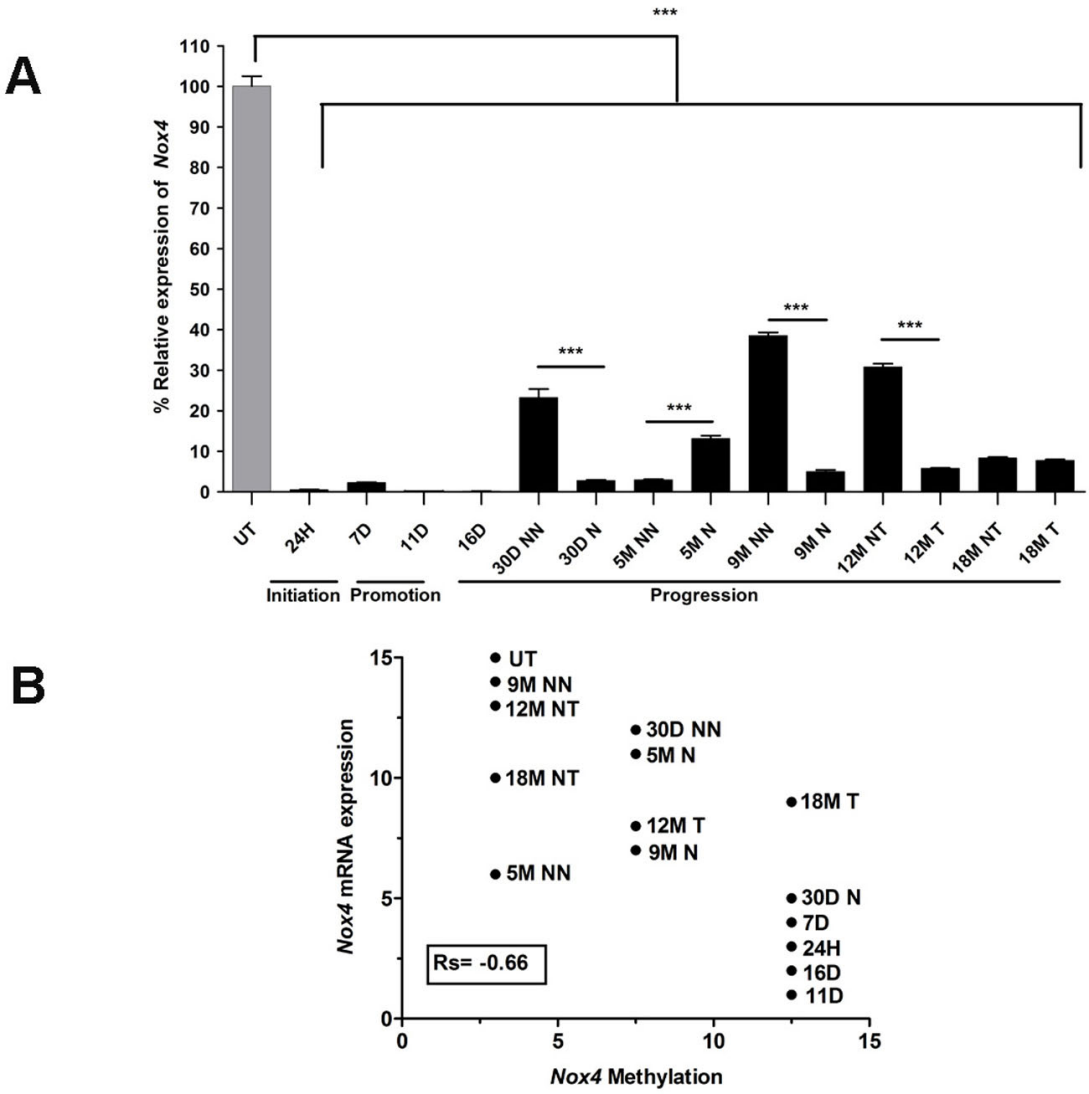
***Nox4* mRNA is downregulated and inversely correlates with its DNA methylation during the initiation, promotion and progression stages of HCG.** (A) Analysis of *Nox4* expression by real-time PCR was performed at 24H, 7D, 11D, 16D, 30D, 5M NN, 5M N, 9M NN, 9M, 12M T, 12M NT, 18M NT and 18M T during liver carcinogenesis. The expression of *Nox4* was normalized to *β-actin* as the loading control gene and was compared with the expression seen in UT samples. The values represent the means±s.d., *n*=3 per group (one-way ANOVA and Bonferroni test, ***P≤0.0001). (B) Spearman's rank correlation between the expression of *Nox4* mRNA and its DNA methylation status by assigning values for expression (15=100% expression in UT samples) and methylation (3=0% methylation, 7.5=0-1% methylation and 12.5=1-10% methylation) during the initiation, promotion and progression stages of HCG. A negative correlation was found (Rs=−0.66). UT, untreated; H, hours; D, days; M, months; NN, non-nodule; N, nodule; NT, non-tumor; T, tumor.

Based on the downregulation of mRNA expression by microarray and the increased methylation pattern observed in *Nox4*, we decided to analyze its expression by qRT-PCR. During the initiation and promotion stages, *Nox4* showed a significant reduction in expression of 97.5% (Fig. 5A; 24H, 7D, and 11D) (***P≤0.0001) compared with UT samples (Fig. 5A; UT). Additionally, all of the analyzed time points were significant with respect to the UT samples during HCG progression; we found more extensive downregulation of *Nox4* at the 30D nodule point (2.75% compared with 23.2% in the 30D non-nodule). This finding reveals a differential downregulation of mRNA between nodules and non-nodules (Fig. 5A; 30D NN vs 30D N) (***P≤0.0001). A similar observation was apparent at 9M nodules, which showed 5% *Nox4* expression compared with 38.56% expression in corresponding non-nodules (Fig. 5A; 9M NN vs 9M N) (***P≤0.0001). At 12 months, the non-tumor (NT) areas also showed differential expression of *Nox4* compared with that in the corresponding tumor samples (30% and 5.8%, respectively) (Fig. 5A; 12M NT vs 12M T) (***P≤0.0001). Non-significant differences were found at 18 months in the non-tumor tissue versus tumor tissue (Fig. 5A; 18M NT vs 18M T), with expression levels of 8.35% and 7.74%, respectively. Collectively, our results show that *Nox4* mRNA is downregulated *in vivo* during all HCG stages.

We used a Spearman's rank correlation test to determine whether there was a relationship between the downregulation of *Nox4* mRNA and the methylation status of its DNA, which was observed in every stage of HCG. The mRNA and DNA samples collected from the non-nodule and the non-tumor areas and the nodule and tumor areas were included in this analysis. Spearman's rank correlation provided a negative result (Rs=−0.66), suggesting that the samples with lower expression levels of *Nox4* mRNA presented higher DNA methylation percentage (Fig. 5B).

### NOX4 protein decreases during the initiation and promotion stages of HCG

**Fig. 6.**
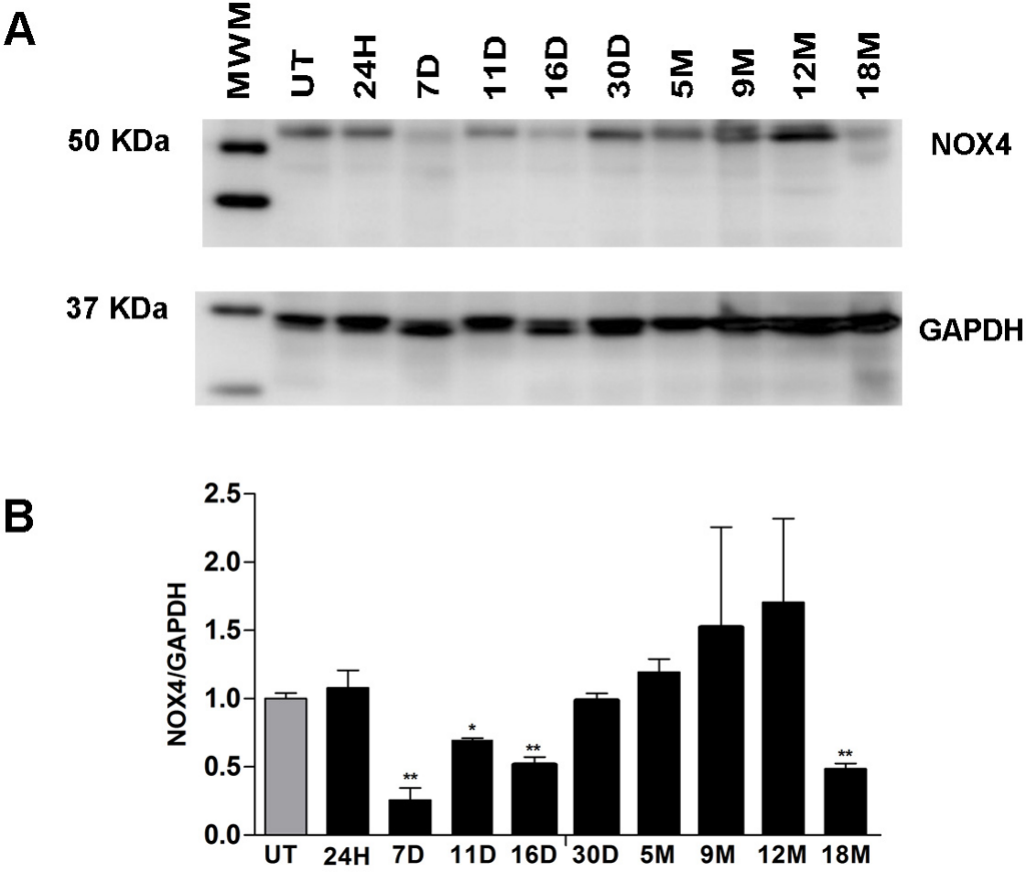
**NOX4 protein is deregulated after the initiation and promotion stages.** (A) Western blot analysis of total protein extracts for NOX4, using GAPDH as the loading control, at 24H, 7D, 11D, 16D, 30D, 5M, 9M, 12M and 18M relative to the UT samples. (B) The graph shows the densitometric analysis at each time point during the development of carcinogenesis in livers. The values represent the means±s.d. of *n*=3 per group (Student's *t*-test with Bonferroni correction at 7, 11 and 16 days and at 18 months, *P≤0.0125, **P≤0.001). MWM, molecular weight marker; UT, untreated; H, hours; D, days; M, months.

**Fig. 7.**
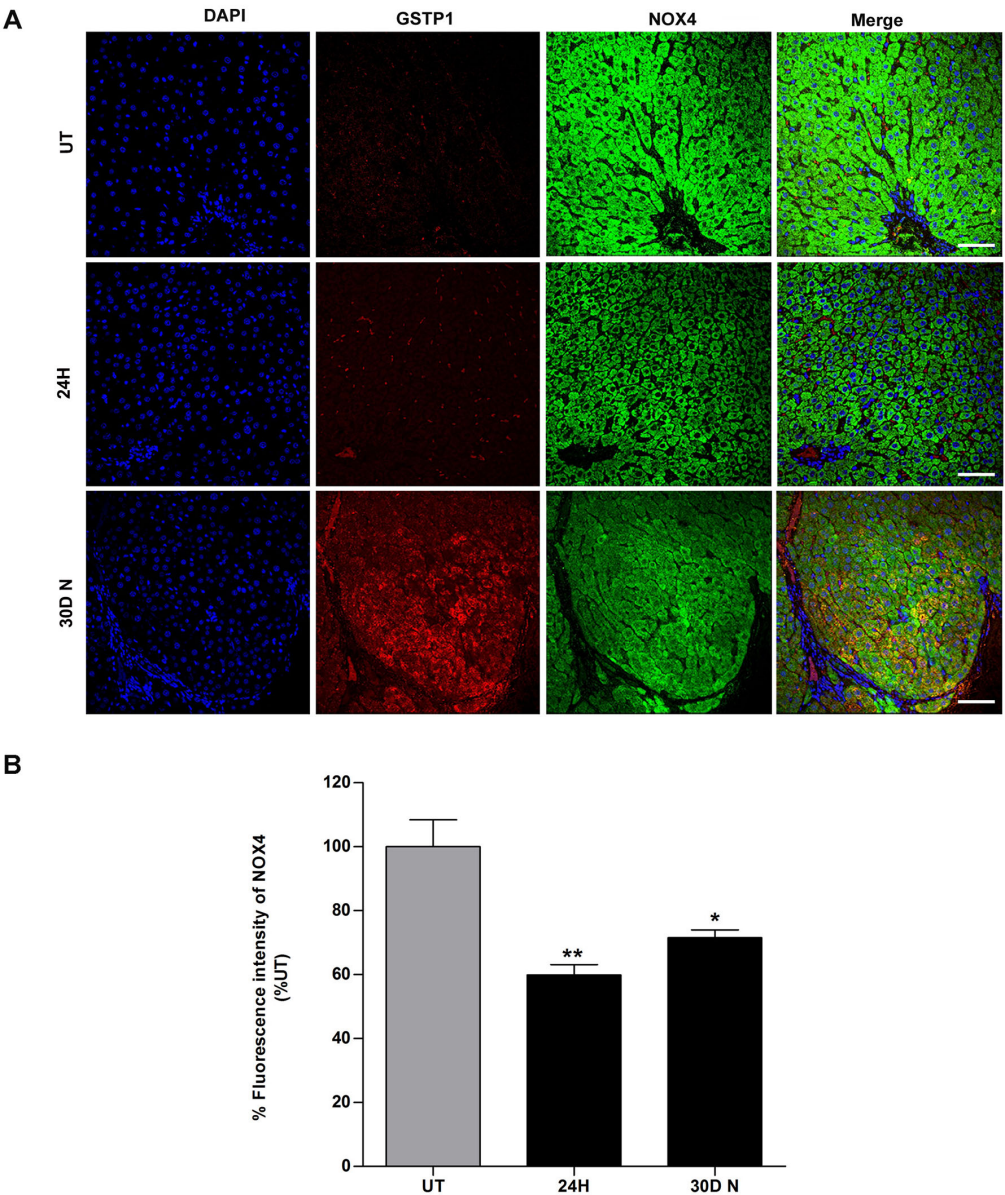
**NOX4 protein levels decrease at 24 h and 30D after initiation.** Double immunofluorescence for GSTP1 and NOX4 was detected by confocal microscopy. (A) UT, 24H and 30D N liver tissues were labeled with DAPI to identify the nuclei (first column). Anti-GSTP1 (second column) and anti-NOX4 (third column) antibodies were used along with secondary antibodies conjugated to the fluorophores TRITC and FITC, and a merged image is shown (last column) for each analyzed time point. Scale bars=50 μm. (B) Graph showing the percentage of fluorescence intensity for detected NOX4 at 24H and 30D N post-treatment with respect to UT liver tissue. The values represent the means±s.d. of *n*=3 per group (one-way ANOVA and Bonferroni test, *P≤0.05, **P≤0.01). UT, untreated; H, hours; D, days; N, nodule.

Some epigenetic studies have associated decreased protein expression with the methylation of CpG islands in the corresponding genes in models of chemical carcinogenesis (Liu et al., 2010). To test this possibility, we analyzed the expression of NOX4 protein by western blotting (Fig. 6A), and we found a significant decrease of 74%, 30% and 48% at 7, 11 and 16 days, respectively (Fig. 6B; 7D, 11D, 16D) (*P≤0.0125, **P≤0.001). During the progression stage, NOX4 displayed a significant decrease of 51% only in the 18-month samples (Fig. 6B; 18M) (**P≤0.001) compared with the UT livers. These results reveal a decrease in the protein levels of NOX4 during the promotion stage just after the administration of 2-AAF and PH. This pattern was also observed during the progression stage at 18 months post-treatment.

### The expression of NOX4 protein decreases in preneoplastic lesions and tumors tissue in rat livers

**Fig. 8.**
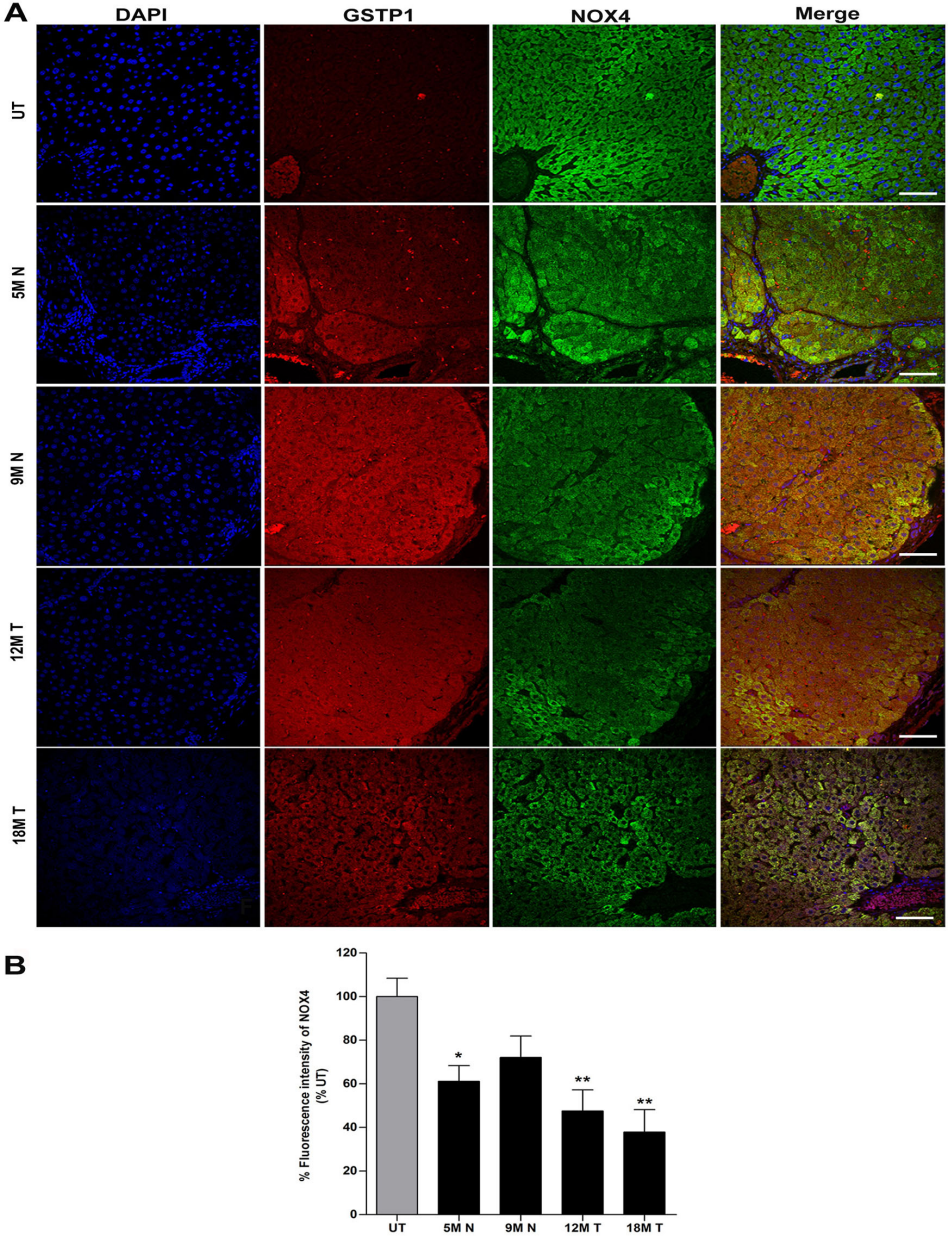
**NOX4 protein is downregulated in preneoplastic lesions and tumors during HCG.** Double immunofluorescence of GSTP1 and NOX4 in paraffin-embedded tissues by confocal microscopy. (A) UT, 5M N, 9M N, 12M T and 18M T liver tissues were labeled with DAPI to visualize nuclei (first column). Anti-GSTP1 (second column) and anti-NOX4 (third column) antibodies were used along with secondary antibodies conjugated to the fluorophores TRITC and FITC, and a merged image is shown (last column) for each analyzed time point. Scale bars=50 μm. (B) Percentage of fluorescence intensity of detected NOX4 at 5M N, 9M N, 12M T and 18M T post-treatment with respect to UT liver tissue. The values represent the means±s.d. of *n*=3 per group (one-way ANOVA and Bonferroni test, *P≤0.05, **P≤0.01). UT, untreated; M, months; N, nodule; T, tumor.

Because the above-described results indicate a downregulation of NOX4 protein in the total extracts compared with that in the UT livers, we decided to further evaluate the expression of NOX4 protein in pre-neoplastic nodules and tumor areas of the livers to confirm our results. To test this, we used double-labeling with glutathione S-transferase pi 1 (GSTP1), a marker of preneoplastic lesions (Maranchie and Zhan, 2005), and NOX4, the protein of interest. The UT liver tissue showed cytoplasmic NOX4 expression and a normal liver tissue architecture (Fig. 7A; UT in NOX4). At 24 h post-DEN treatment, NOX4 showed a significant decrease (Fig. 7A; 24H in NOX4) of 40% compared with its expression in UT liver tissue (Fig. 7B). At 30 days, we observed a clear demarcation of the preneoplastic lesion that was positive for GSTP1 (Fig. 7A; 30D N in GSTP1) and that displayed a 28.5% decrease in NOX4 compared with the expression level seen in corresponding UT tissues (Fig. 7B). We also observed preneoplastic lesions and tumor tissue that were positive for GSTP1 (Fig. 8A; 5M N, 9M N, 12M T and 18M T in GSTP1), which simultaneously exhibited decreases in the expression of NOX4 (Fig. 8A; 5M N, 9M N, 12M T and 18M T in NOX4) of 38%, 27%, 52% and 59%, respectively (Fig. 8B) compared with that in UT liver tissue (Fig. 8A; UT in NOX4). Thus, our data show that NOX4 protein expression decreases *in vivo* in preneoplastic lesions and tumor tissue. Fig. S5 shown for a better appreciation of the difference in NOX4 protein expression between non-nodular, nodular, tumor and non-tumor areas.

## DISCUSSION

In this paper, we report a model of HCG that features the downregulation of different genes containing CpG islands. We found that in the carcinogen-treated animals, the *Nox4* gene exhibits a methylation pattern that differs from untreated animals. The methylation of the *Nox4* gene correlates with the downregulation of mRNA and protein.

The use of the present animal model is supported by the feasibility of obtaining samples to analyze the changes that occur during the carcinogenic process, which could enhance our understanding of the molecular mechanisms involved in the pathogenesis of HCG and HCC. These HCG models have been used extensively to detail GEP during different stages of carcinogenesis (Oliveira et al., 2007; Pogribny and Rusyn, 2014; Teoh et al., 2015; Vasquez-Garzon et al., 2015).

Previously, our group used a bioinformatics analysis of an Affymetrix microarray of GEP, and we found that 1248 genes were downregulated at very early stages (24 h) and up to 18 months post-treatment in this HCG model. Based on these data, we selected a set of four deregulated genes (*Casp3*, *Cldn1*, *Pex11a* and *Nox4*) with known biological functions (Honda et al., 2007; Schrader et al., 2012; Weyemi et al., 2013; Yakovlev et al., 2010).

In addition, in carcinogenic processes and HCG models, changes occur in the epigenetic landscape through the abnormal expression of DNA methyltransferases (DNMT), global genomic hypomethylation and gene-specific DNA hypermethylation in tumor suppressor genes or in genes involved in a variety of important cellular pathways (Kulis and Esteller, 2010; Pogribny et al., 2008; Pogribny and Beland, 2013). Previous work using this model of HCG revealed the deregulation of DNMT, which is responsible for establishing and maintaining the CpG methylation patterns in normal cells, suggesting that changes in DNA methylation patterns may contribute to the progression of liver cancer (Valencia Antunez et al., 2014).

In this study, we examined the methylation profiles of *Casp3*, *Cldn1*, *Pex11a* and *Nox4*, focusing on the CpG islands in the promoter regions of these genes. *Nox4* was the only gene that exhibited changes in methylation patterns relative to untreated rats. In this model of liver carcinogenesis, *Casp3*, *Cldn1* and *Pex11a* expression levels did not appear to be regulated by methylation in their CpG islands. However, we cannot rule out the contribution of other epigenetic mechanisms such as post-translational modifications of histones. Some reports have shown that *Casp3*, some claudins and *DUOX1* and *DUOX2* are regulated by epigenetic mechanisms during maturation of the rat brain, as well as in ovarian cancer cells and lung cancer (Honda et al., 2007; Luxen et al., 2008; Yakovlev et al., 2010).

Through a sodium bisulfite conversion DNA sequencing assay, we found five methylated CpG sites in the *Nox4* gene promoter at 7 and 11 days post-diethylnitrosamine and 2-acetylaminofluorene (Fig. 4B; 7D and 11D) with respect to untreated rats. Low levels of DNA methylation in CpG islands can be associated with gene silencing in the absence of hypermethylation at the gene promoter. Even the methylation of one CpG dinucleotide can directly interfere with transcription factor binding, resulting in gene silencing. This phenomenon has been previously reported for several transcription factors, such as AP-2, cMyc/Myn and E2F NF-B, among others (Singal and Ginder, 1999). Therefore, our data suggest that the methylation of these five CpG dinucleotides is related to a decrease in *Nox4* gene expression in the HCG model.

The five methylated CpG dinucleotides are located in the end part of the *Nox4* CpG island (Fig. 4A). These regions are called CpG island ‘shores’ and have recently been described to have low CpG density compared with the CpG islands themselves, and which are located near traditional CpG islands. Some reports have shown that the main mechanism of gene silencing by DNA methylation is unrelated to methylation in the CpG island core. It has been proposed that these regions (CpG island ‘shores’) exhibit hypomethylation and significantly affect gene expression (Doi et al., 2009; Rao et al., 2013).

We propose that the downregulation of *Nox4* is associated with its DNA methylation status, as determined by a non-parametric method. The samples displaying reduced expression of *Nox4* showed a higher percentage of methylation. Interestingly, *Nox4* mRNA exhibited dramatic downregulation during the initiation stage immediately after the first carcinogenic stimulus with DEN at 24 h (30-fold decrease in the microarray and a 97.5% decrease by qRT-PCR) relative to the UT rats, and this event persisted throughout the promotion and progression stages. Also, immunofluorescence analysis showed that NOX4 protein was located in the cytoplasm of the untreated liver tissues and was downregulated in the pre-neoplastic nodules and tumor tissues. These data demonstrate clear deregulation of NOX4 protein throughout the HCG process.

*Nox4* belongs to the NADPH oxidase family, the specific physiological function of which is to generate ROS (Krause, 2004; Weyemi et al., 2013). The ROS generated by members of this family have been implicated in numerous biological functions (Ushio-Fukai, 2006). Studies from our lab and others have shown that increases in ROS play an important role in carcinogenesis, mainly during the initiation stage. Although a principal function of *Nox4* is to produce ROS, this gene was downregulated in the present study. The oxidative stress measured by lipid peroxidation during HCG was not related to the expression of *Nox4* but was a product of the carcinogenic treatment with DEN and 2-AAF as well as the proliferative stimulus with PH, at 10, 11 and 16 days (Fig. S4).

The results suggest that Nox4 does not participate in the formation of ROS or alter their production in this model. For this reason, we suggested that other molecules could participate in the production of ROS to compensate for the lack of *Nox4*. Based in a gene expression analysis during HCG previously reported (Vasquez-Garzon et al., 2015), we identified the overexpression of other molecules, including *Nox2*, during the development of liver carcinogenesis G.S.L.-A. and S.V.-T., unpublished data). *Nox2* belong to the NADPH oxidase family and plays an important role in cellular processes, such as the stimulation of tumor angiogenesis, and its overexpression has been previously reported in human prostate and gastric cancers (Wang et al., 2015).

The deregulation of the NAPDH oxidase family is involved in carcinogenesis (Jiang and Torok, 2014; Weyemi et al., 2013). Overexpression of *NOX4* has been demonstrated in various cancers (Kanda et al., 2015; Lee et al., 2007; Maranchie and Zhan, 2005; Yamaura et al., 2009) and in animal models, concomitant with the development of spontaneous fibrosis induced in genetically modified mouse models and with CCl_4_ (Sancho et al., 2012).

However, recent reports have described the downregulation of *Nox4* in DEN-induced rat livers (Liu et al., 2009). A recent report has shown the downregulation of *Nox4* in mice after PH during tumorigenesis induced by DEN; the same report showed that silencing *Nox4* in xenograft experiments in athymic mice conferred an advantage to human hepatocarcinoma cells, as shown by more rapid tumor formation and growth. These authors suggested that NOX4 plays an essential role as a tumor suppressor in liver tissue (Crosas-Molist et al., 2014).

Our data, together with previous findings, suggest that *Nox4* participates in HCG, beginning at early stages of the process. In this study, we showed specific methylation of the CpG Island in the promoter of *Nox4*. Moreover, we corroborated this observation with a preliminary analysis of liver cancer cells (HepG2), in which methylation of the CpG island of the *Nox4* promoter was evident. Interestingly, the extent of methylation in HepG2 cells was even higher than that found in the DNA of rat livers subjected to carcinogenic treatment.

Few reports have focused on the epigenetic mechanisms that regulate the downregulation of the NAPDH oxidase family. For example, research has shown that the promoters of *DUOX1* and *DUOX*2 are methylated, which correlates with their downregulation at the protein level in lung cancer (Luxen et al., 2008). Regarding *Nox4*, which was the only gene to display promoter methylation in our study, previous studies have reported regulation by histone deacetylases (HDACs) in human umbilical vein endothelial cells (HUVECs) (Siuda et al., 2012). In a similar context, the observation that microRNA *miR-25* may contribute to the negative regulation of *Nox4* has been reported in diabetic nephropathy (Fu et al., 2010). Also, NOX4 is regulated in a non-replicative senescence model in lung fibroblasts via epigenetic mechanisms, specifically by post-translational changes in histones associated with the promoter region of the gene. These authors also observed methylation in the *Nox4* promoter, and they found different amounts of methylated and unmethylated copies in senescent and non-senescent cells. Ultimately, the role of methylation in this model is not clear (Sanders et al., 2015).

For a better understanding of the mechanisms that regulate the expression of *Nox4* during the HCG, other epigenetic modifications, such as post-translational changes in histones, should be explored. Additionally, transcription factors may bind to regions containing the CpG islands of *Nox4* or microRNAs, which might help explain the downregulation of *Nox4* in this model. Future studies will be required to investigate the behavior of *Nox4* expression in other types of cancers. The downregulation of *Nox4* during early stages of carcinogenesis likely favors tumor development. Our result also warrants the exploration of methods to reverse the methylation of the *Nox4* promoter as a possible cancer therapy.

Based on the present findings, we conclude that the DNA methylation of the CpG island *Nox4*, as well as the corresponding downregulation of this gene at the mRNA and protein levels, are linked to the development of liver cancer in this model. Because these alterations in the mRNA and protein expression levels are not observed in untreated rats, *Nox4* may function as a tumor suppressor in liver.

## MATERIALS AND METHODS

### Hepatocarcinogenesis rat model

For this study, we used two-month-old male Fischer-344 rats (*Rattus norvegicus*) weighing 180-200 g. The rats were obtained from the Production Unit of Experimental Laboratory Animals (UPEAL-CINVESTAV, Mexico City, Mexico), and they were given unlimited access to water and were fed a standard laboratory animal diet (PMI Feeds Inc., Laboratory Diet, Richmond, IN, USA). All experiments were performed according to the Institutional Animal Care and Use Committee guidelines. Initially, fifty rats were divided into ten groups and were subjected to a resistant hepatocyte model of HCG (Semple-Roberts et al., 1987) with modifications (Marche-Cova et al., 1995). For tumor initiation, the rats were intraperitoneally injected with 200 mg/kg of DEN (Sigma, St. Louis, MO, USA) dissolved in distilled water. At 7, 8 and 9 days, 20 mg/kg of 2-AAF was administered orally to the rats. On day 10 after initiation, the rats were subjected to a partial hepatectomy (PH). All rats were anesthetized and euthanized by exsanguination. The livers were collected at different points post-treatment: 0 h (untreated rats), 24 h (24H, referred to as the initiation stage), 7 and 11 days (7D and 11D, referred to as the promotion stage), 16 and 30 days and 5, 9, 12 and 18 months (16D, 30D, 5M, 9M, 12M and 18M, referred to as the progression stage) (Fig. 1B). In this study, we used groups of five rats for each HCG time point. Because 20-30% of the animals died during the carcinogen treatment, we only analyzed the data from three rats per group to ensure different liver samples for all experiments. The collected livers were stored at −80°C until the extraction of gDNA, mRNA and total protein. Additional portions of livers were fixed in formaldehyde, which were embedded in paraffin.

### DNA extraction

DNA was extracted from each rat liver sample at 24 h, at 7, 11, 16 and 30 days and at 5, 9, 12 and 18 months post-treatment. We obtained DNA from nodules at 30 days and at 5 and 9 months. We also obtained DNA from tumor tissue at 12 and 18 months. The DNA purification was performed using a Wizard Genomic DNA Purification kit (Promega, Madison, WI, USA). Briefly, 60 mg of liver and cell pellets from the HepG2 cell line growing at 80% confluence were homogenized with a nuclear lysis solution, incubated at 65°C for 30 min and then incubated at 37°C for 30 min with 3 µl of RNase A (20 mg/ml, Invitrogen, Carlsbad, CA, USA). The proteins were precipitated, and DNA was precipitated from the supernatant with isopropanol followed by a wash with 70% ethanol. The samples were eluted in molecular biology grade H_2_O and then quantified.

### Bisulfite conversion and methylation status analysis

All of the DNA samples extracted above were sodium bisulfite-treated using an EZ DNA Methylation-Gold Bisulfite conversion kit (Zymo Research, Alameda, CA, USA) according to the manufacturer's protocol. 500 ng of DNA were used as the input for each sample.

### Methylation status analysis

MS-HRM technology was used to simultaneously determine the methylation status in a qualitative and rapid manner for the DNA samples. This technology is based on a comparison between the HRM profiles of the PCR products for the screened samples and the HRM profiles of the PCR products obtained from a mixture of artificially methylated and unmethylated standards (Wojdacz, 2012). In the methylation analysis, the CpG sites in a DNA strand of a certain length can present with three possibilities: samples with all CpG sites methylated, samples with all CpG sites devoid of methylation and heterogeneous methylation, in which the samples display different amounts of CpG site methylation in different alleles (Wojdacz, 2012).

The experiments were designed and performed according to a standard protocol for this technique (Wojdacz et al., 2008). A Light Cycler 480 High-Resolution Master Mix (Roche, Indianapolis, IN, USA) was used for PCR amplification in conjunction with specific primers for a region of the CpG islands located within the promoters of *Casp3*, *Cldn1*, *Pex11a* and *Nox4* in rats and *NOX4* in human (Fig. S1). The experiments were controlled against a range of standards consisting of 100%, 10%, 1% and 0% mixes of methylated rat DNA with unmethylated background DNA (EpigenDx, Hopkinton, MA, USA). Moreover, we used universal controls of human methylated and unmethylated DNA for the analysis of HepG2 cells (EpigenDx). PCR amplification, HRM profiling and data analyses were performed using a LightCycler 480 II instrument (Roche, Penzberg, Germany).

### Sequencing analyses

A subset of MS-HRM PCR products was sequenced using the Sanger method, which has been previously described (Wojdacz et al., 2010). The PCR products were sequenced using the same primers as those in the MS-HRM analysis. The forward strand was sequenced two times from the representative samples of UT, 7 and 11 days post-treatment.

### Analysis of gene expression by qRT-PCR

TriPure reagent (Roche, Mannheim, Germany) was used for RNA extraction from liver tissues collected from UT rats at 24 h, at 7, 11, 16 and 30 days and at 5, 9, 12 and 18 months post-treatment. At 30 days, 5 months and 9 months, we obtained mRNA from non-nodule and nodule areas, and at 12 and 18 months, we obtained mRNA from non-tumor and tumor areas. cDNA synthesis was performed using an oligo dT 12-18 bp primer and SuperScript II reverse transcriptase (Invitrogen). Maxima SYBR Green/ROX qPCR Master Mix (2X) (Thermo Scientific, Waltham, MA USA) was used in the RT-PCR experiments, which were run on a StepOnePlus Real-Time PCR System (Applied Biosystems, Waltham, MA USA). The primers used were as follows: *Nox4*, forward 5′-ctgtacaaccaagggccagaa-3′ and reverse 5′-tgcagttgaggttcaggacaga-3′ (Meng et al., 2008); *β-actin*, forward 5′-cctctatgccaacacagtgc-3′ and reverse 5′-catcgtactcctgcttgctg-3′. The annealing temperature was 61°C for both genes. The 2ΔΔCT method was used to calculate relative changes in the expression of *Nox4* and *β-actin* (Livak and Schmittgen, 2001).

### Western blotting

Total protein extracts were obtained from frozen rat livers collected during the initiation, promotion and progression stages. All samples were homogenized in lysis buffer (100 mM Tris-HCl pH 7.4, 1.5 M NaCl, Triton X-100) containing a protease inhibitor cocktail tablet (Roche, Mannheim, Germany) and then centrifuged at 1100 ***g*** for 15 min at 4°C. The supernatant was collected and centrifuged at 15,000 ***g*** for 10 min at 4°C. The protein concentration was determined by BCA assay (bicinchoninic acid assay) using Bio-Rad protein assay reagent (Bio-Rad, Hercules, CA, USA). Thirty micrograms of protein were mixed with 2× Laemmli sample buffer (Bio-Rad) and boiled at 95°C for 5 min. The proteins were separated by SDS-PAGE on a 12% gel and then transferred to a PVDF membrane for immunoblotting analysis (Amersham Hybond-P, GE Healthcare, Buckinghamshire, UK). The membranes were blocked for 1 h in TBS-Tween 20 and 10% nonfat milk. Subsequently, the membranes were incubated with peroxidase-conjugated rabbit monoclonal anti-NOX4 (1:5000, Abcam, Cambridge, MA, USA) and mouse monoclonal anti-GAPDH (1:10,000, Cell Signaling, Danvers, MA, USA) antibodies overnight at 4°C. The membranes were incubated with goat anti-rabbit IgG-HRP and rabbit anti-mouse IgG-HRP secondary antibodies (Sigma-Aldrich, St. Louis, MO, USA) at 1:15,000 dilutions. The signal was visualized with Immobilon Western Chemiluminescent HRP Substrate Reagent (Millipore Corporation, Billerica, MA, USA) and developed using C-DiGit equipment (LI-COR, NE, USA). The densitometric values were obtained and normalized to 1 with respect to the UT rats.

### NOX4 and GSTP1 immunofluorescence

For double immunolabeling of GSTP1 and NOX4, the liver tissues collected at 24 h, 30 days and 5, 9, 12 and 18 months were cut into 4-μm thick sections, deparaffinized, permeabilized in PBS with 0.2% Triton X-100 and treated for 1 min with 0.1% Sudan Black B to reduce tissue autofluorescence. Subsequently, all sections were blocked for 1 h at room temperature with 5% BSA and then incubated with goat polyclonal anti-GSTP1 (Santa Cruz Biotechnology, sc-134469, CA, USA) and rabbit monoclonal anti-NOX4 (Abcam, Ab109225, Cambridge, MA, USA) antibodies at dilutions of 1:100. The sections were incubated with goat IgG and rabbit IgG secondary antibodies, which were tagged with tetramethylrhodamine-isothiocyanate (TRITC) and fluorescein-isothiocyanate (FITC) (Jackson ImmunoResearch, PA, USA), respectively, in PBS for 2 h at room temperature at dilutions of 1:200. Nuclei were stained with DAPI (4′, 6-diamino-2-phenylindole, Molecular Probes, MA, USA) at a 1:5000 dilution. As a control for antibody specificity, UT liver tissue was incubated with a rabbit primary antibody isotype control (Invitrogen, Frederick, MD, USA) (data not shown). The liver tissues were analyzed by confocal microscopy using a Leica TCP-SP8 confocal laser-scanning microscope (Leica Microsystems, Heidelberg, Germany) at 40× magnification (N.A., 1.3) with an HC Plan-Apochromat CS2 oil immersion lens. The mean fluorescence intensity of each captured image was analyzed with Leica LAS-AF Lite 2.X software, and the data were plotted.

### Statistical analysis

Statistical analyses of qRT-PCR, immunofluorescence and lipid peroxidation were performed using a one-way analysis of variance (ANOVA) and Bonferroni's multiple comparison test (*P*≤0.05). For western blotting, Student's *t*-test was performed with Bonferroni correction for samples at 7, 11 and 16 days and at 18 months (*P*≤0.0125). The experiments were performed using three rats for each analyzed time point and they were repeated three times independently during HCG, and the values are expressed as the means±s.d. to assess significant differences between the groups. To relate the expression of *Nox4* mRNA to the methylation percentage during HCG, we used the non-parametric Spearman's rank method, which revealed a negative correlation (Rs=−0.66).

Spearman correlation is a non-parametric test that measures the correlation or association between two variables, providing ρ values between −1 and +1, which indicate a negative or a positive association (Myers and Sirois, 2006).
